# Supplementary material for: Current Knowledge and Perceptions of Bariatric Surgery among Diabetologists and Internists in Poland
Source: J Clin Med. 2022 Apr 5;11(7):2028. doi: 10.3390/jcm11072028 (PMC8999568; doi:10.3390/jcm11072028)
Supplement: Supplementary file 1 [file jcm-11-02028-s001.zip › jcm-1658990-supplementary.pdf]

Knowledge and perceptions of bariatric surgery among Polish diabetologists and internists - survey

*Required

Good morning. Thank you very much for participating in the study we have prepared. We invite you to fill out the form addressed to doctors dealing with treatment of diabetic diseases. Please remember that your answers will be entered into the database only if the form is completed in full.

If you have any questions, please contact the study coordinators:

Prof. Piotr Major MD PhD
II Department of Surgery, Jagiellonian University Medical College
e-mail: piotr.major@uj.edu.pl

Assoc. Prof. Magdalena Szopa, MD, PhD
Department of Metabolic Diseases, Jagiellonian University Medical College
e-mail: magdalena.szopa@uj.edu.pl

Thank you for your participation, we wish you luck in filling out the form!

Demographics

1. Please choose your gender. *

   Mark only one oval.

   - Male
   - Female
   - Other: ____________

2. Please type your age in years. *

   ____

3. Please choose your specialty. *

   Tick all that apply.

   - [ ] Diabetologist Internist
   - [ ] Diabetologist in training
   - [ ] Internist in training

   Other: [ ] ____________

4. Please type your time in clinical practice in years. *

   ____

5. Please choose your workplace. *

Tick all that apply.

- [ ] Primary healthcare Outpatient
- [ ] specialist care
- [ ] Non-university affiliated hospital
- [ ] University affiliated hospital

Other: [ ] ______________________

6. Please choose the number of patients you see monthly. *

Mark only one oval.

- ( ) <50
- ( ) 50-100
- ( ) 100-150
- ( ) 150-200
- ( ) >200

7. Please type the number of morbidly obese patients you see monthly. *

____

8. Please type the number of morbidly obese patients suffering from diabetes you see monthly. *

____

9. How often do you refer morbidly obese patients to a surgical consultation for bariatric surgery?

Mark only one oval.

	1	2	3	4	5	
I never refer to consultation	( )	( )	( )	( )	( )	I refer each morbidly obese patient to consultation

Self-estimated knowledge, attitudes and beliefs regarding bariatric surgery

10. Please assess your knowledge about qualification for the surgical treatment of obesity. *

Mark only one oval.

	1	2	3	4	5	
I know nothing about it	( )	( )	( )	( )	( )	I have an excellent knowledge about it

11. Please assess your knowledge about preparing patients for bariatric surgery. *

Mark only one oval.

	1	2	3	4	5	
I know nothing about it	( )	( )	( )	( )	( )	I have an excellent knowledge about it

12. Please assess your knowledge about the principles and scope of multidisciplinary teams caring for bariatric patients. *

Mark only one oval.

	1	2	3	4	5	
I know nothing about it						I have an excellent knowledge about it

13. Please assess your knowledge about basic types of surgical procedures in the field of bariatric surgery and their mechanisms. *

Mark only one oval.

	1	2	3	4	5	
I know nothing about it						I have an excellent knowledge about it

14. Please assess your knowledge about long-term consequences and possible health problems in a group of patients after bariatric surgery. *

Mark only one oval.

	1	2	3	4	5	
I know nothing about it						I have an excellent knowledge about it

15. Please assess your knowledge about principles of diabetes treatment in patients admitted to hospital for bariatric surgery. *

Mark only one oval.

	1	2	3	4	5	
I know nothing about it						I have an excellent knowledge about it

16. I have access to the appropriate tools to deal with morbidly obese patients (e.g., scales >150 kg, beds, diagnostic equipment). *

Mark only one oval.

- Strongly disagree
- Disagree
- I don't know/I have no opinion
- Agree
- Strongly agree

17. Vitamins and microelements should be supplemented in all patients after bariatric surgery *

Mark only one oval.

- Strongly disagree
- Disagree
- I don't know/I have no opinion
- Agree
- Strongly agree

18. Bariatric surgery is an effective treatment for metabolic syndrome *

Mark only one oval.

- Strongly disagree
- Disagree
- I don't know/I have no opinion
- Agree
- Strongly agree

19. Bariatric surgery has a better effect on glycemic control than an intensive conservative treatment *

Mark only one oval.

- Strongly disagree
- Disagree
- I don't know/I have no opinion
- Agree
- Strongly agree

Knowledge about bariatric surgery

20. Which adult patients are eligible for bariatric surgery? *

Tick all that apply.

- [ ] Patients with BMI equal of above 40 kg/m2
- [ ] Patients with BMI equal of above 35 kg/m2
- [ ] Patients with BMI in range of 35-40 kg/m2 and in whom weight loss after bariatric surgery may improve course of obesity-related comorbidities
- [ ] Patients with BMI in range of 30-35 kg/m2 and in whom weight loss after bariatric surgery may improve course of obesity-related comorbidities
- [ ] I don't know

21. What are absolute contraindications to performing bariatric surgery in adult patients? *

Tick all that apply.

- [ ] Lactation
- [ ] Severe coagulation disorders Active
- [ ] alcohol or drug addiction
- [ ] Inability to take part in long-term follow-up after surgery
- [ ] I don't know.

22. Are patients below 18 years old eligible for bariatric surgery? *

Mark only one oval.

- ( ) Yes
- ( ) No
- ( ) I don't know

23. What is the most frequently performed type of bariatric surgery in Poland? *

Mark only one oval.

- ( ) Vertical banded gastroplasty (VBG)
- ( ) Laparoscopic sleeve gastrectomy (LSG)
- ( ) Biliopancreatic diversion with duodenal switch (BPDDS)
- ( ) Roux-en-Y gastric bypass (RYGB)
- ( ) I don't know

24. Please choose three of the most important tests to be performed before bariatric surgery. *

Tick all that apply.

- [ ] Diurnal glycemic profile
- [ ] Arterial blood gases Complete
- [ ] blood count Urinalysis
- [ ] Serum alanine and aspartate aminotransferase Serum
- [ ] sodium, potassium
- [ ] APTT, INR, bleeding time
- [ ] Chest X-ray
- [ ] Eye fundus examination

Other: [ ] ______________________

25. Which metabolic control criteria are an indication for postponing planned bariatric surgery? *

Tick all that apply.

- [ ] Blood glucose >250 mg/dl (13,9 mmol/l)
- [ ] HbA1c > 8,5%
- [ ] Glycosuria and acetonuria
- [ ] Blood glucose >180 mg/dl (10 mmol/l)
- [ ] HbA1c > 7%
- [ ] I don't know

26. What is the proper peri-operative blood glucose range in patients undergoing bariatric surgery? *

Mark only one oval.

- 100–180 mg/dl
- 80-100 mg/dl
- 120-150 mg/dl
- 100-200 mg/dl

27. What is the 30-day mortality after bariatric surgery? *

Mark only one oval.

- 0,1-0,3%
- 2-6%
- 10-15%
- 20-25%
- I don't know

28. What is the recommended scheme for outpatient follow-up after bariatric surgery? *

Mark only one oval.

- 1 month after surgery, then every 3 month during first year after surgery, and finally every 1 year 1 and
- 6 months after surgery, then every 2 years
- 1 month after surgery, and then every 6 months
- 1 month after surgery, then every 3 months during first year after surgery, and finally every 6 months
- Other scheme

29. What is the criteria of diabetes and comorbidities resolution in patients in whom treatment was stopped? *

Tick all that apply.

- [ ] HbA1c < 6,5%
- [ ] The patient does not have episodes of hypoglycaemia Total
- [ ] cholesterol <4 mmol/l and LDL <2 mmol/l
- [ ] Weight loss >15% in relation to weight at qualification for surgery Blood
- [ ] pressure <140/90 mmHg
- [ ] I don't know

30. Is contraception recommended for women after bariatric surgery? *

Mark only one oval.

- ( ) Contraception is not necessary after bariatric surgery.
- ( ) Yes, at least 6 months after surgery
- ( ) Yes, at least 1 year after surgery
- ( ) Yes, at least 2 years after surgery
- ( ) I don't know.

Summary

31. I am interested in broadening my knowledge of bariatric surgery. *

Mark only one oval.

- ( ) Strongly disagree
- ( ) Disagree
- ( ) I don't know/I have no opinion
- ( ) Agree
- ( ) Strongly agree

32. Which issues about bariatric surgery would be most interesting to you? *

Tick all that apply.

- [ ] Rules of qualifying patients for bariatric surgery
- [ ] Knowledge of the types of bariatric surgeries
- [ ] The effectiveness of bariatric surgeries
- [ ] Principles of reimbursement of bariatric surgeries by the National Health Fund
- [ ] Location of bariatric surgery centers
- [ ] Guidelines for long-term follow-up of patients after bariatric surgery
- [ ] Other: ____________
